# Successful mRNA COVID-19 vaccination in a patient with a history of severe polyethylene glycol anaphylaxis

**DOI:** 10.1186/s13223-022-00698-8

**Published:** 2022-06-20

**Authors:** Daniel H. Li, Erika Lee, Christine Song

**Affiliations:** Division of Clinical Immunology and Allergy, Department of Medicine, University of Toronto, Toronto, ON Canada

**Keywords:** COVID-19, mRNA vaccine, Anaphylaxis, Excipient testing, Polyethylene glycol

## Abstract

**Background:**

The mechanism of action behind anaphylactic reactions to the mRNA COVID-19 vaccines remains unknown, but the excipient polyethylene glycol, PEG-2000, has been implicated. Initial recommendations were made for excipient testing with PEG-3350 to help risk stratify individuals and identify an etiology. Here we present a case of a patient with a history of polyethylene glycol anaphylaxis and positive skin testing to PEG-3350, who successfully received both doses of the Pfizer-BioNTech COVID-19 mRNA vaccine in a single step with only premedication.

**Case presentation:**

A 56-year-old man was referred to our clinic for assessment of his eligibility in receiving the COVID-19 vaccine given a history of anaphylaxis to PEG. He had two anaphylactic episodes: one in 2018 to methylprednisolone acetate intra-articular injection and one to oral PEG-3350 in 2020. Confirmatory skin prick testing was done in our clinic to PEG-3350 that was positive at 35 mm with appropriate positive and negative controls. Despite this he wanted to receive the PEG-containing mRNA COVID-19 vaccines and was counselled on the risks and benefits. He successfully received both doses of the Pfizer-BioNTech COVID-19 mRNA vaccine in a single step with only pre-treatment with Cetirizine 20 mg daily and Montelukast 10 mg daily for 5 days.

**Conclusions:**

In conclusion, our case demonstrates that a patient with a confirmed polyethylene glycol anaphylaxis could safely receive both doses of the COVID-19 mRNA vaccines in a single step with pre-treatment. We hope that our case will further support the limited role in skin testing to PEG in the assessment of COVID-19 mRNA vaccine allergy and highlight the need for further research to elucidate the mechanism of action behind these allergic reactions.

## Background

Almost immediately after the Emergency Use Authorization (EUA) by the Food and Drug Administration of the Pfizer-BioNTech COVID-19 mRNA vaccine and Moderna COVID-19 mRNA vaccine, initial reports of anaphylaxis occurred [1]. Formal guidance was developed by the National Advisory Committee on Immunization (NACI) in Canada for the administration of these vaccines. The most recent NACI guidelines recommend that the COVID-19 vaccine should not be routinely offered to individuals who are allergic to any components of the vaccine and list polyethylene glycol (PEG) as a potential allergen in both the Pfizer-BioNTech COVID-19 mRNA and Moderna COVID-19 mRNA vaccines. [2] The etiology of anaphylaxis to mRNA COVID-19 vaccines remains unknown, but the excipient polyethylene glycol, PEG-2000, has been implicated and excipient testing with PEG-3350 was initially recommended to help risk stratify individuals and identify an etiology [3, 4]. Here we present a case of a patient with a history of PEG anaphylaxis and positive skin testing to PEG-3350, who successfully received both doses of the Pfizer-BioNTech COVID-19 mRNA vaccine in a single step with only premedication.

## Case presentation

A 56-year-old man was referred to our clinic for assessment of his eligibility in receiving the COVID-19 vaccine given a history of anaphylaxis to PEG. His first reaction occurred in June 2018 when he received a methylprednisolone acetate (Depo-Medrol^®^) intra-articular injection for osteoarthritis. Within five minutes of receiving the injection, he developed flushing, diffuse pruritus, shortness of breath, wheezing, chest pain and palpitations. He also had a syncopal episode with documented hypotension (63/40 mmHg) and hypoxia and was transported to the Emergency Department for further management. In 2020, he went on to have an anaphylactic reaction to oral PEG-3350 (Restoralax^®^), where he developed diffuse pruritis, urticaria, diaphoresis, shortness of breath and wheeze within ten minutes of taking his first dose. He was treated with intramuscular epinephrine and had resolution of his symptoms within four hours.

Given the history of PEG anaphylaxis, skin prick testing (SPT) with PEG-3350 was done as the next step. Wheal and erythema were measured at 15 min. He tested positive (35 mm in diameter) on PEG-3350 (68 mg/ml) with an appropriate response to histamine for positive control and normal saline for negative control. However, despite his clinical history of anaphylaxis and positive skin prick testing to PEG-3350, he wanted to receive the PEG-containing mRNA COVID-19 vaccine due to the high rates of COVID-19 in the community at that time. He was counseled on the risks and understood that a confirmed PEG allergy was considered a contraindication.

The patient received pre-treatment with Cetirizine 20 mg daily and Montelukast 10 mg daily for 5 days. Then, we administered the first dose of the Pfizer-BioNTech COVID-19 mRNA vaccine to him in a single step in our hospital-based allergy clinic. He did not develop any subjective or objective evidence of an allergic reaction within the hour of observation. Three weeks later he received his second dose of the Pfizer-BioNTech COVID-19 mRNA vaccine in a single step at a community vaccine clinic with the same pre-treatment course without any adverse reactions.

## Discussion and conclusions

Initial case reports of PEG allergy as the etiology of anaphylaxis to the mRNA vaccines suggested an IgE-mediated mechanism [4]. However, there is increasing evidence suggesting that this may not be the case. Warren et al. looked at 11 patients, with a history of reported anaphylaxis to the mRNA vaccine and found that none had a positive skin prick test to PEG or had PEG IgE detected. However, 91% had positive basophil activation results to PEG and high levels of IgG to PEG were detected [5]. Warren et al. postulated that the mechanism of anaphylaxis to the mRNA vaccines may be due to an IgG complement activation-related pseudoallergy (CARPA) [5]. This may suggest why our patient with IgE-mediated sensitization to PEG confirmed on skin prick test was able to tolerate both doses of the mRNA vaccine.

Other studies have also provided support in the limited role of excipient testing. Wolfson et al. recently looked at 80 individuals with reported allergic reactions after the first dose of a mRNA COVID-19 vaccine and found 75% of these individuals were able to receive the second dose regardless of skin test results (the positive skin testing was only to intradermal methylprednisolone acetate and not PEG-3350) [5]. More recently a case series of 12 patients was published by Picard et al. that looked at the safety of COVID-19 vaccination in patients with PEG allergy, unfortunately only 1 out of their 12 patients had a history of anaphylaxis to PEG, skin test positive to PEG 3350 and went on to receive an mRNA COVID-19 vaccine in one step [7]. We are reporting one of the first few cases of a patient who successfully received the mRNA vaccine in a single step with positive epicutaneous testing to PEG-3350 and a history of severe PEG anaphylaxis.

The limitation of our observation is that we are unable to discern if our patient was only able to successfully receive the mRNA COVID-19 vaccine in a single step because of premedication or being PEG allergic does not translate into a high risk of reacting to mRNA COVID-19 vaccines. We suspect that it is the latter, as in the case series by Picard et al. they had one patient who was PEG allergic, skin test positive and received the COVID-19 mRNA vaccine in a single step without premedication. [7]

There is currently no standardized approach to PEG allergic patients receiving COVID-19 vaccines given the paucity of cases and the differences in approaches between different centers. When faced with the challenge of safely facilitating COVID-19 vaccination in patients with PEG allergy, different centers have utilized different strategies including premedication, split dosing, and use of a non-mRNA COVID-19 vaccine.

We hope that our case will further support the limited role in skin testing to PEG in the assessment of COVID-19 mRNA vaccine allergy and contribute to our repertoire of cases so that eventually a standardized guideline can be generated to help allergists manage these patients coming to us for COVID-19 vaccine with a history of PEG allergy.

## Data Availability

All data generated or analysed during this study are included in this published article.

## Abbreviations

EUA: Emergency Use Authorization
NACI: National Advisory Committee on Immunization
mRNA: Messenger ribonucleic acid
PEG: Polyethylene glycol
SPT: Skin prick testing
IgE: Immunoglobulin E
IgG: Immunoglobulin G
CARPA: Complement activation-related pseudoallergy

## References

[1] Gee J, Marquez P, Su J, Calvert GM, Liu R, Myers T (2021). First month of COVID-19 vaccine safety monitoring—United States, December 14, 2020-January 13, 2021. MMWR Morb Mortal Wkly Rep.

[2] Canada Go. Recommendations on the use of COVID-19 vaccines https://www.canada.ca/en/public-health/services/immunization/national-advisory-committee-on-immunization-naci/recommendations-use-covid-19-vaccines.html#b9. Accessed 2 Nov 2021.

[3] Banerji A, Wickner PG, Saff R, Stone CA, Robinson LB, Long AA (2021). mRNA vaccines to prevent COVID-19 disease and reported allergic reactions: current evidence and suggested approach. J Allergy Clin Immunol Pract.

[4] Sellaturay P, Nasser S, Islam S, Gurugama P, Ewan PW (2021). Polyethylene glycol (PEG) is a cause of anaphylaxis to the Pfizer/BioNTech mRNA COVID-19 vaccine. Clin Exp Allergy.

[5] Warren CM, Snow TT, Lee AS, Shah MM, Heider A, Blomkalns A (2021). Assessment of allergic and anaphylactic reactions to mRNA COVID-19 vaccines With confirmatory testing in a US regional health system. JAMA Netw Open.

[6] Wolfson AR, Robinson LB, Li L, McMahon AE, Cogan AS, Fu X (2021). First-dose mRNA COVID-19 vaccine allergic reactions: limited role for excipient skin testing. J Allergy Clin Immunol Pract.

[7] Picard M, Drolet JP, Masse MS, Filion CA, Faisel A, Fein M (2022). Safety of COVID-19 vaccination in patients with polyethylene glycol allergy: a case series. J Allergy Clin Immunol Pract.

